# Human Amnion-Derived Mesenchymal Stem Cells Promote Osteogenic Differentiation in Human Bone Marrow Mesenchymal Stem Cells by Influencing the ERK1/2 Signaling Pathway

**DOI:** 10.1155/2016/4851081

**Published:** 2015-11-30

**Authors:** Yuli Wang, Fei Jiang, Yi Liang, Ming Shen, Ning Chen

**Affiliations:** Jiangsu Key Laboratory of Oral Diseases, Nanjing Medical University, No. 140, Han Zhong Road, Nanjing, Jiangsu 210029, China

## Abstract

Human amnion-derived mesenchymal stem cells (HAMSCs) are considered to be an important resource in the field of tissue engineering because of their anti-inflammatory properties and fewer ethical issues associated with their use compared with other sources of stem cells. HAMSCs can be obtained from human amniotic membranes, a readily available and abundant tissue. However, the potential of HAMSCs as seed cells for treating bone deficiency is unknown. In this study, HAMSCs were used to promote proliferation and osteoblastic differentiation in human bone marrow mesenchymal stem cells (HBMSCs) in a Transwell coculture system. Proliferation levels were investigated by flow cytometry and immunofluorescence staining of 5-ethynyl-2′-deoxyuridine (EdU). Osteoblastic differentiation and mineralization were evaluated in chromogenic alkaline phosphatase (ALP) activity substrate assays, Alizarin red S staining, and RT-PCR analysis of early HBMSCs osteogenic marker expression. We demonstrated that HAMSCs stimulated increased alkaline phosphatase (ALP) activity, mRNA expression of osteogenic marker genes, and mineralized matrix deposition. Moreover, the effect of HAMSCs was significantly inhibited by U0126, a highly selective inhibitor of extracellular signaling-regulated kinase 1/2 (ERK1/2) signaling. We demonstrate that HAMSCs promote osteogenic differentiation in HBMSCs by influencing the ERK1/2 signaling pathway. These observations confirm the potential of HAMSCs as a seed cell for the treatment of bone deficiency.

## 1. Introduction

An emerging medical and socioeconomic problem among patients requiring dental implants is bone volume inadequacy, which increases the difficulty of restoring oral function. Recently, tissue engineering using suitable seed cells has shown great potential in the treatment of bone deficiency. Human bone marrow mesenchymal stem cells (HBMSCs), osteoblasts (OB), and dental pulp stem cells (DPSCs) have been used as seed cells [1–3], but most have disadvantages, such as high immunogenicity and limited availability.

Human amniotic membrane (AM) is a readily available and highly abundant tissue composed of a single layer of epithelial cells, underlying fibroblasts, and an avascular collagenous stroma [4]. AM has been shown to promote epithelization, reduce inflammation, and prevent scarring [5, 6]. Human amnion-derived mesenchymal stem cells (HAMSCs) are associated with low anti-inflammatory properties and fewer ethical issues than other sources of stem cells, thus providing considerable benefits as seed cells in bone tissue engineering [7]. Recent studies showed that although HAMSC osteogenesis was much lower than other marrow mesenchymal stem cells' [8], the acellular amniotic membrane matrix was capable of enhancing osteogenic differentiation in DPSCs by activating ERK1/2 signaling [9], which led us to hypothesize that the function of HAMSCs in tissue-engineered bone is derived from its effect on other cells. In this study, a Transwell coculture system was used to determine the in vitro effects of HAMSCs on osteogenic differentiation in HBMSCs. Interestingly, we found that HAMSCs stimulated increased levels of alkaline phosphatase activity (ALP), mRNA expression of osteogenic marker genes, and mineralized matrix deposition, thus confirming that HAMSCs are capable of providing a preferential environment for driving osteogenic differentiation in HBMSCs.

Previous studies have revealed that extracellular signaling-regulated kinase 1/2 (ERK1/2), a member of the mitogen-activated protein kinase (MAPK) family, regulates the differentiation and proliferation of HBMSCs [10–12]. Moreover, the transcriptional activity of runt-related transcription factor 2 (Runx2) is essential for subsequent bone formation and osteoblast differentiation. Runx2 activity plays an important role in controlling the expression of osteogenic genes, including osteocalcin (OC) and ALP [10, 11, 13], which are activated by ERK1/2 signaling [14, 15]. In this study, we further investigated the role of the ERK1/2 signaling pathway in osteogenic differentiation in HBMSCs cocultured with HAMSCs.

## 2. Material and Methods

### 2.1. Chemicals and Reagents

The HBMSC cell line PTA-1058 was obtained from the American Type Culture Collection (ATCC, Manassas, VA, USA). Phosphate-buffered saline (PBS), fetal bovine serum (FBS), *α*-minimum essential medium (*α*MEM), trypsin-EDTA, and penicillin G-streptomycin sulfate were purchased from Gibco Life Technologies. The dimethyl sulfoxide (DMSO), sodium hydroxide (NaOH), para-nitrophenyl phosphate (pNPP), *β*-glycerophosphate, ascorbic acid, and dexamethasone were purchased from Sigma-Aldrich. Transwells (6-Well Millicell Hanging Cell Culture Inserts, 0.4 *μ*m, PET) and 6-well culture plates were purchased from Millipore (Bedford, MA, USA). The TRIzol reagent and polymerase chain reaction (PCR) primers were purchased from Invitrogen (Carlsbad, CA, USA), and reverse transcriptase- (RT-) PCR kits were purchased from TaKaRa Bio (Otsu, Japan). The antibodies specific for human CD105, CD90, CD79b, CD44, CD29, and Runx2 were purchased from R&D Systems (Boston, MA, USA). The Cell-Light EdU Apollo 488 In Vitro Imaging Kit was purchased from RiboBio (Guangzhou, China). The goat anti-rabbit and anti-mouse IgG, phospho-p44/42 MAPK rabbit mAb (p-ERK1/2), and p44/42 MAPK (ERK1/2) rabbit mAb were purchased from Cell Signaling Technology (Danvers, MA, USA). The protein assay kit, Alizarin red S (pH 4.4), bicinchoninic acid (BCA) assay kit, phenylmethane sulfonyl-fluoride, RIPA buffer, and DAPI were purchased from Beyotime (Shanghai, China).

### 2.2. Cell Culture

**Figure 1:**
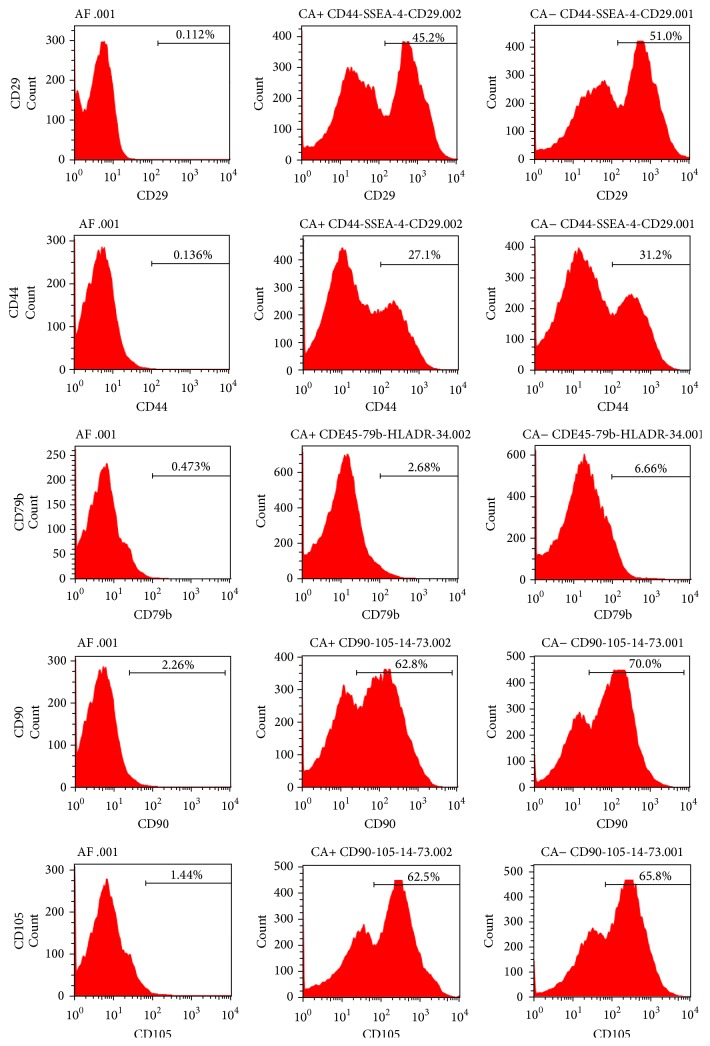
Characterization of third-passage adherent amniotic cells: cells were stained with monoclonal antibodies specific for human CD105, CD90, CD79b, CD44, and CD29 and analyzed by flow cytometry.

HAMSCs were collected from discarded amniotic membrane samples obtained within 24 h using the pancreatin/collagenase digestion method as described previously [16]. Third-passage adherent amniotic cells were collected from a flask using ethylenediaminetetraacetic acid (EDTA) treatment and characterized by flow cytometric analysis (BD Biosciences, Franklin Lakes, NJ, USA) of staining with monoclonal antibodies specific for human CD105, CD90, CD79b, CD44, and CD29 [17, 18] (Figure 1). After characterization, HAMSCs (5 × 10^4^ cells/cm^2^) were seeded into 60 mm plates in *α*MEM supplemented with 10% FBS, 100 U/L penicillin, and 100 mg/L streptomycin in a humidified atmosphere of 5% CO_2_ at 37°C. Cells from passages 3–5 were used in this study and culture medium was changed every 3 days. The experiments have been approved by the Ethics Committee of Nanjing Medical University. Informed consent was obtained before all participants enrolled in this study.

### 2.3. Preparation of the Coculture System

A Transwell coculture system was used to investigate the effects of HAMSCs on proliferation and osteogenic differentiation of HBMSCs. HBMSCs were seeded at an initial cell density of 5 × 10^4^ cells/cm^2^ in 6-well culture plates. Transwells were placed in other 6-well culture plates and HAMSCs were seeded at increasing HBMSCs : HAMSCs ratios (1 : 1, 1 : 2, and 1 : 3) as 5 × 10^4^ cells/cm^2^, 10 × 10^4^ cells/cm^2^, and 15 × 10^4^ cells/cm^2^ in the Transwells. After cells were attached (approximately 24 h), Transwells containing HAMSCs were moved into the corresponding wells of the 6-well culture plate containing HBMSCs to create the HAMSC/HBMSCs Transwell coculture system. HBMSCs in wells without Transwells served as the control groups, while HBMSCs with Transwells were designated as the treatment groups.

### 2.4. Proliferation Assay

The effect of HAMSCs on HBMSCs proliferation was measured at 3, 6, and 12 d by flow cytometry and immunofluorescence staining of 5-ethynyl-2′-deoxyuridine (EdU) [19]. Briefly, Transwells containing HAMSCs were moved into the corresponding wells of the 6-well culture plate containing HBMSCs in routine culture media supplemented with 10% FBS for 24 h. After starvation in serum-free medium for 24 h, the medium was replaced with culture medium containing 10% FBS. HBMSCs were then harvested at 3, 6, and 12 d and fixed with 75% ice-cold ethanol at 4°C for 30 min in the dark [20]. DNA content was measured by a FACScan flow cytometer (BD Biosciences, USA). Cell cycle fractions (G0, G1, S, and G2 M phases) were determined by flow cytometry. After 12 days in the presence of medium containing 10% FBS, Transwells containing HAMSCs were removed and a Cell-Light EdU Apollo 488 In Vitro Imaging Kit was used to investigate the EdU expression levels in each group. Briefly, 100 *μ*L EdU medium (50 *μ*M) was added to each well. After incubation for 2 h, the EdU medium was removed and HBMSCs were washed twice with PBS and fixed with 75% ethanol. After lysing with Triton X-100 (0.5%) for 15 min, staining solution was added to each well and incubated for 30 min. DAPI was used to stain the cell nuclei. EdU expression levels were examined by immunofluorescence staining and the area of stained EdU-positive nodules relative to the total culture surface was measured by Image-Pro Plus (IPP) analysis. Ten images were captured for each well, and the mean percentage was calculated.

### 2.5. In Vitro Osteogenic Differentiation

Transwells containing HAMSCs were moved into the corresponding wells in the 6-well culture plate containing HBMSCs after attachment. The regular medium in all cultures (including the controls) was replaced with osteogenic medium (OS) containing 10 mM *β*-glycerophosphate, 100 nM ascorbic acid, and 100 nM dexamethasone when the cells approached confluence after 3-4 days in culture. The mRNA expression levels of ALP, Runx2, and OC, which are related to osteoblastic (OB) differentiation and considered to be early HBMSCs osteogenic markers [21–23], were analyzed by RT-PCR on day 7. HBMSCs cocultured with HAMSCs were also subjected to investigation of ALP activity and Western blotting analysis of ERK1/2 signaling as well as Runx2 expression. U0126, a highly selective inhibitor of ERK1/2 signaling, was prepared in DMSO and used in the signaling inhibition assay at the concentration of 20 mM as previously described [9]. To eliminate the influence of U0126 on HAMSCs, HBMSCs were cocultured with HAMSCs for 7 days before removal of the Transwells containing HAMSCs and treatment with U0126 for 24 h. Moreover, the ERK1/2 signaling in the long-term culture was investigated by treating the cells with U0126 for 3 days after removing the Transwells.

### 2.6. RNA Isolation and RT-PCR

Total cellular RNA was isolated from HBMSCs in the control and treatment groups using TRIzol according to the manufacturer's instructions. The mRNA was reverse-transcribed into cDNA by using a PrimeScript RT Master Mix kit. Real-time reverse-transcription PCRs were performed with SYBR Premix Ex Taq kit and ABI 7300 Real-Time PCR System (Foster City, CA, USA). The primers used were as follows: human ALP (forward, 5′-TGGAGGTTCAGAAGCTCAACACCA-3′; reverse, 5′-ATCTCGTTCTCTGAGTACCAGTC-3′), human Runx2 (forward, 5′-CCGCACAACCGCACCAT-3′; reverse, 5′-CGCTCCGGCCCACAAATCTC-3′), human OC (forward, 5′-CAGCGGTGCAGAGTCCAGCAAA-3′; reverse, 5′-GATGTGGTCAGCCAACTCGTCA-3′), and human glyceraldehyde-3-phosphate dehydrogenase (GAPDH, forward, 5′-GGGCTGCTTTTAACTCTGGT-3′; reverse, 5′-GCAGGTTTTTCTAGACGG-3′); GAPDH was used as the internal control. Gene expression was calculated using the 2^−ΔΔCt^ method [20, 24].

### 2.7. ALP Activity and Mineralized Matrix Formation

After the end of the 7-day coculture period, Transwells containing HAMSCs were removed. HBMSCs in each group were then washed twice with PBS and lysed with Triton X-100 (0.5%) for 15 min. A bicinchoninic acid (BCA) assay kit was used to determine the protein concentration [25, 26]. Para-nitrophenyl phosphate (pNPP, Sigma) was used as the substrate and the cells were incubated at 37°C for 30 min with gentle shaking in the dark. The reaction was terminated by the addition of 3 N sodium hydroxide solution (NaOH, Sigma-Aldrich) and ALP activity was determined from the absorbance at 405 nm as previously described [27–29]. The enzyme activity was expressed as micromoles of reaction product per minute per total protein.

HBMSCs cocultured with HAMSCs for 14 days were stained with Alizarin red S to assess mineralized matrix deposition for bone nodule formation [27, 30]. Cells were fixed with 75% dehydrated alcohol after washing twice with PBS and then stained with 40 mM Alizarin red S (pH 4.4) for 5 min at room temperature. Red staining of mineralized matrix deposition was observed using a microscope and the area of stained extracellular matrix relative to the total culture surface was measured by Image-Pro Plus (IPP) analysis. Ten images were captured for each well, and the mean percentage was calculated.

### 2.8. Western Blotting Analysis

After the end of the 7-day coculture period, Transwells containing HAMSCs were removed. HBMSCs in each group were lysed in RIPA buffer containing 1 mM phenylmethane sulfonyl-fluoride according to the manufacturer's instructions. The total protein concentration was determined using a bicinchoninic acid (BCA) assay kit. Protein lysates (20 *μ*g) were separated by sodium dodecyl sulfate-polyacrylamide gel electrophoresis (SDS-PAGE) and then transferred onto 0.22 *μ*m polyvinylidene difluoride membranes (Millipore, Bedford, Mass, USA). After blocking, membranes were incubated overnight at 4°C with specific antibodies for the detection of Runx2 (1 : 1,000), ERK1/2 (1 : 500), and p-ERK1/2 (1 : 500). After three washes with PBST (0.5% Tween 20 in PBS), the membranes were incubated with the relevant secondary antibodies (1 : 1,000) for 1 h at 37°C, washed, and visualized with an ECL detection kit (Amersham Pharmacia, New Jersey, USA). *β*-actin (1 : 500) served as an internal control.

### 2.9. Statistical Analysis

Representative data are presented as the mean and standard deviation (SD) of at least three independent samples. Data were analyzed using one-way analysis of variance (ANOVA). *P* values < 0.05 were considered indicating statistical significance.

## 3. Results and Discussion

### 3.1. HBMSC Proliferation in the Transwell Coculture System

**Figure 2:**
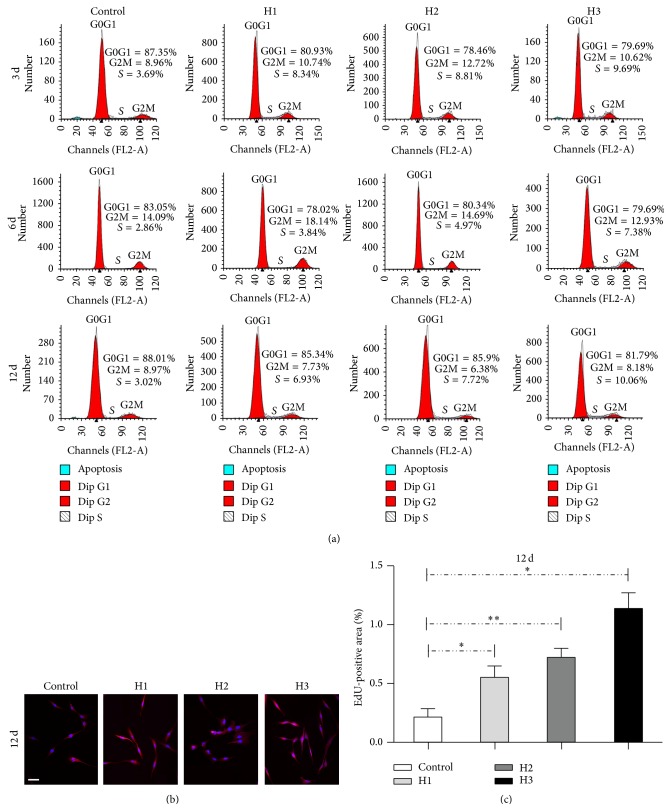
The effect of HAMSCs on HBMSC proliferation was measured by flow cytometry and immunofluorescence staining of 5-ethynyl-2′-deoxyuridine (EdU). (a) Cell cycle fractions (G0, G1, S, and G2 M phases) at 3, 6, and 12 d. (b) Immunofluorescence staining of EdU at 12 d. (c) Area of stained EdU-positive nodules relative to the total culture surface at 12 d was measured by Image-Pro Plus (IPP) analysis. Scale bar: 100 *μ*m, ^*∗*^ *P* < 0.05, and ^*∗∗*^ *P* < 0.01. Control: HBMSCs cultured without HAMSCs; HBMSCs cocultured with HAMSCs; H1: HBMSC : HAMSC = 1 : 1; H2: HBMSC : HAMSC = 1 : 2; H3: HBMSC : HAMSC = 1 : 3.

The proliferation of HBMSCs seeded in the Transwell coculture system was analyzed by flow cytometry (Figure 2(a)) and immunofluorescence staining of 5-ethynyl-2′-deoxyuridine (EdU) as a cell proliferation marker (Figure 2(b)). Cell cycle fractions (G0, G1, S, and G2 M phases) were determined by flow cytometry at 3, 6, and 12 d. The S-phase checkpoints increased slightly with the HBMSC : HAMSC ratio; however, immunofluorescence staining of EdU at 12 d revealed a statistically significant increase of EdU-positive HBMSC nodules with the HBMSC : HAMSC ratio in coculture (treatment groups) compared to the single-culture (control) groups (Figure 2(c)). This demonstrated that HBMSC proliferation was accelerated in the Transwell coculture system.

### 3.2. Expression of Osteogenic Marker Genes

**Figure 3:**
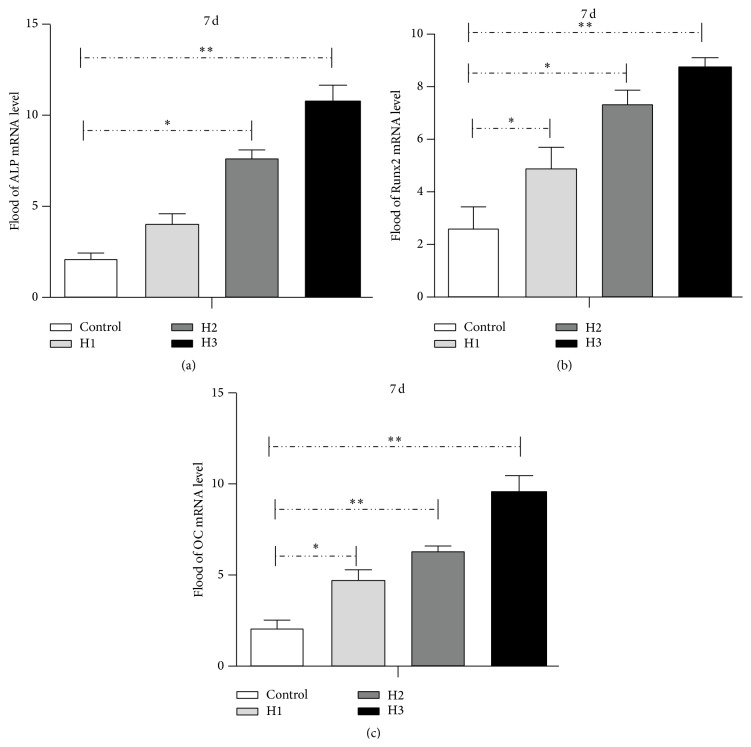
RT-PCR analysis of HBMSCs cultured with or without HAMSCs. Transwells containing HAMSCs were moved into the correlating wells containing HBMSCs in regular medium after cells were attached. After the cells approached confluence in regular medium, both cell types were cultured in OS medium for 7 days. The mRNA expression of ALP, Runx2, and OC was analyzed by RT-PCR. GAPDH was used as the internal control. (a) Alkaline phosphatase (ALP); (b) runt-related transcription factor 2 (Runx2). (c) Osteocalcin (OC). ^*∗*^ *P* < 0.05; ^*∗∗*^ *P* < 0.01. Control: HBMSCs cultured without HAMSCs; HBMSCs cocultured with HAMSCs; H1: HBMSC : HAMSC = 1 : 1; H2: HBMSC : HAMSC = 1 : 2; H3: HBMSC : HAMSC = 1 : 3.

After attachment of HAMSCs, Transwells were moved into the corresponding wells containing HBMSCs in regular medium. Both cell types were exposed to OS medium when cellular growth approached confluence. Gene expression of the early HBMSCs osteogenic markers ALP, Runx2, and OC was analyzed by RT-PCR after osteogenic induction (Figure 3). After 7 days, greater upregulation of ALP, Runx2, and OC mRNA expressions was detected in the treatment groups compared to that in the control groups; this increase correlated with the increase in HBMSC : HAMSC ratio.

### 3.3. ALP Activity and Mineralized Matrix Formation

**Figure 4:**
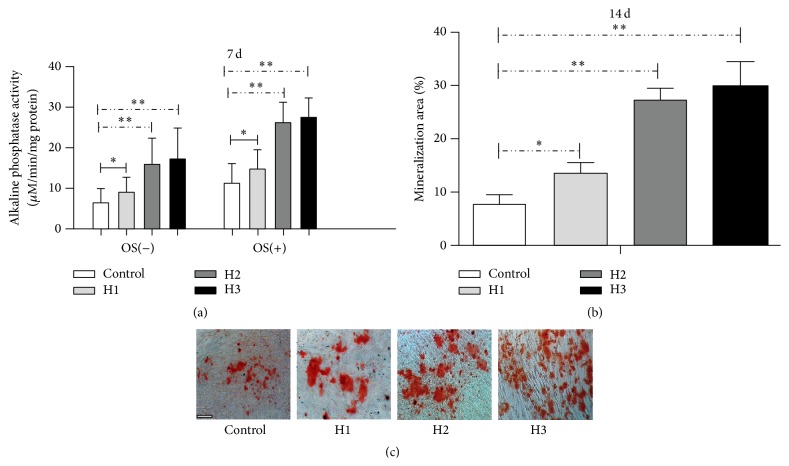
ALP activity and mineralized matrix deposition in HBMSCs cultured with or without HAMSCs. Transwells containing HAMSCs were moved into the correlating wells containing HBMSCs in regular medium after cells were attached. After the cells approached confluence in regular medium, both cell types were cultured in OS medium for 7 days and 14 days. (a) ALP activity was measured at 7 d using p-nitrophenyl phosphate as the substrate. (b) Mineralized matrix deposition was measured at 14 d by Alizarin red S staining and Image-Pro Plus (IPP) analysis. The relative intensity is expressed as a percentage of the stained extracellular matrix area relative to the total culture surface area. (c) Micrographs of mineralized matrix deposition, scale bar: 100 *μ*m. ^*∗*^ *P* < 0.05; ^*∗∗*^ *P* < 0.01. Control: HBMSCs cultured without HAMSCs; HBMSCs cocultured with HAMSCs; H1: HBMSC : HAMSC = 1 : 1; H2: HBMSC : HAMSC = 1 : 2; H3: HBMSC : HAMSC = 1 : 3.

We examined ALP activity after 7 days in culture with and without OS to evaluate the effect of HAMSC on osteoblastic differentiation of HBMSCs in the coculture system [31]. Compared to the control groups, ALP activity increased significantly in the treatment groups with the HBMSC : HAMSC ratio, regardless of the presence or absence of OS (Figure 4(a)).

Figure 4(b) shows the percentage of the HBMSC culture surface stained positively for extracellular matrix in the presence of OS. Higher levels of mineralization were observed in the treatment groups after 14 days of culture compared to that in the control groups, and the percentage of mineralized nodules increased with the HBMSC : HAMSC ratio (Figure 4(c)). These observations indicate that HAMSC positively influences the mineralization of HBMSC in the in vitro coculture system.

### 3.4. Effect of HAMSCs on ERK1/2 Phosphorylation and Runx2 Protein Level

**Figure 5:**
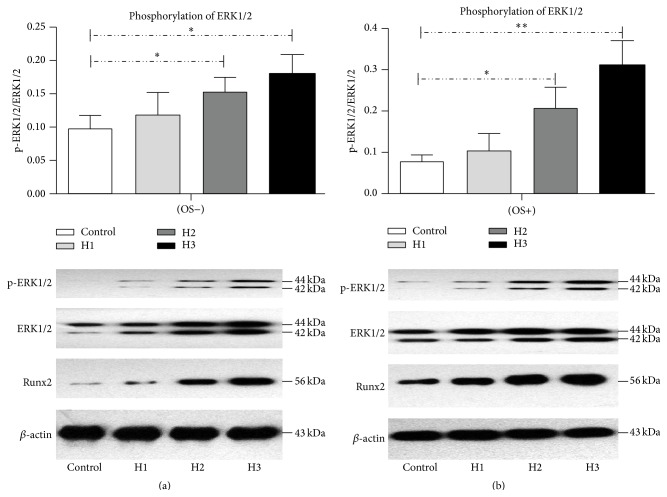
Expression of p-ERK1/2, ERK1/2, and Runx2 proteins in HBMSCs cultured with or without HAMSCs. Transwells containing HAMSCs were moved into the correlating wells containing HBMSCs in regular medium after cells were attached. After the cells approached confluence in regular medium, both cell types were cultured in OS medium for 7 days. (a) Cells were treated without OS for 7 days. Densitometric measures of the band intensity are expressed as the signal ratios indicating the level of ERK phosphorylation (p-ERK1/2/ERK1/2). (b) Cells were treated with OS for 7 days. Densitometric measures of the band intensity are expressed as the signal ratios indicating the level of ERK phosphorylation (p-ERK1/2/ERK1/2). ^*∗*^ *P* < 0.05; ^*∗∗*^ *P* < 0.01. Control: HBMSCs cultured without HAMSCs; HBMSCs cocultured with HAMSCs; H1: HBMSC : HAMSC = 1 : 1; H2: HBMSC : HAMSC = 1 : 2; H3: HBMSC : HAMSC = 1 : 3.

It is well-known that ERK1/2, a member of the MAPK family, regulates the differentiation, mineralization, and proliferation of HBMSCs [10–12]. Moreover, the transcriptional activity of Runx2 is essential for subsequent bone formation and osteoblast differentiation. Therefore, we investigated the effect of HAMSCs on ERK1/2 signal activation in HBMSCs. Figures 5(a) and 5(b), respectively, show ERK1/2 phosphorylation and Runx2 protein level in HBMSCs in the control and treatment groups after 7 days in culture with and without OS. OS increased ERK1/2 phosphorylation and Runx2 protein level in HBMSCs cocultured with HAMSCs. Furthermore, this effect was increased with the HBMSC : HAMSC ratio regardless of the presence or absence of OS. These results suggested that both OS and HAMSCs enhance ERK1/2 phosphorylation and Runx2 protein level, which might play a role in regulating HBMSC differentiation and mineralization.

**Figure 6:**
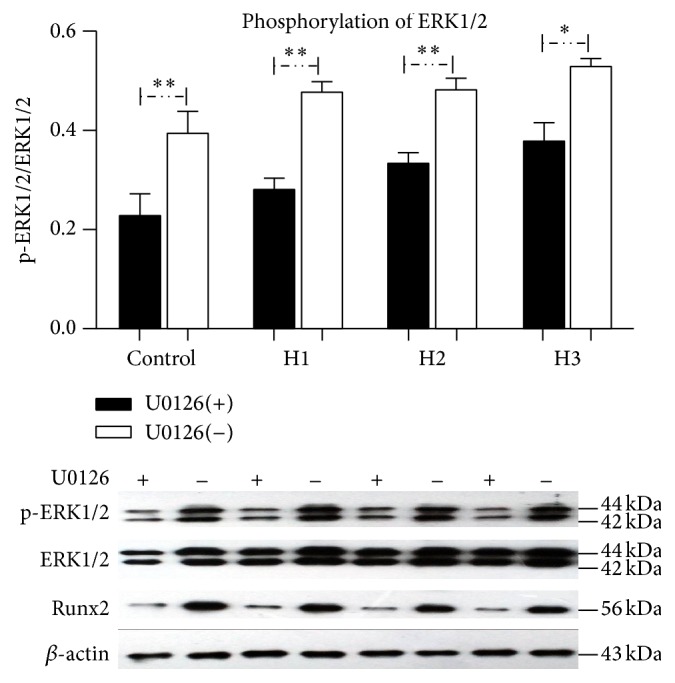
U0126 inhibited the expression of p-ERK1/2, ERK1/2, and Runx2 proteins in HBMSCs cultured with or without HAMSCs. Transwells containing HAMSCs were moved into the correlating wells containing HBMSCs in regular medium after cells were attached. After the cells approached confluence in regular medium, both cell types were cultured in OS medium for 7 days, followed by treatment with and without U0126 for a further 24 h. Densitometric measures of the band intensity are expressed as the signal ratios indicating the level of ERK phosphorylation (p-ERK1/2/ERK1/2), ^*∗*^ *P* < 0.05; ^*∗∗*^ *P* < 0.01. Control: HBMSCs cultured without HAMSCs; HBMSCs cocultured with HAMSCs; H1: HBMSC : HAMSC = 1 : 1; H2: HBMSC : HAMSC = 1 : 2; H3: HBMSC : HAMSC = 1 : 3.

The effect of HAMSCs on ERK1/2 signaling pathway activation was further explored using U0126, a highly selective inhibitor of ERK1/2. HBMSCs in the control and treatment groups were treated with U0126 for a further 24 h after 7 days in culture with mineralizing medium as described previously [9]. To eliminate the influence of U0126 on HAMSCs, the Transwells were removed at the end of 7-day culture with OS medium. ERK1/2 phosphorylation and Runx2 expression were significantly decreased in HBMSCs in a manner consistent with the effects of U0126 (Figure 6).

### 3.5. Inhibitory Effects of U0126 on HBMSCs Differentiation and Mineralization

**Figure 7:**
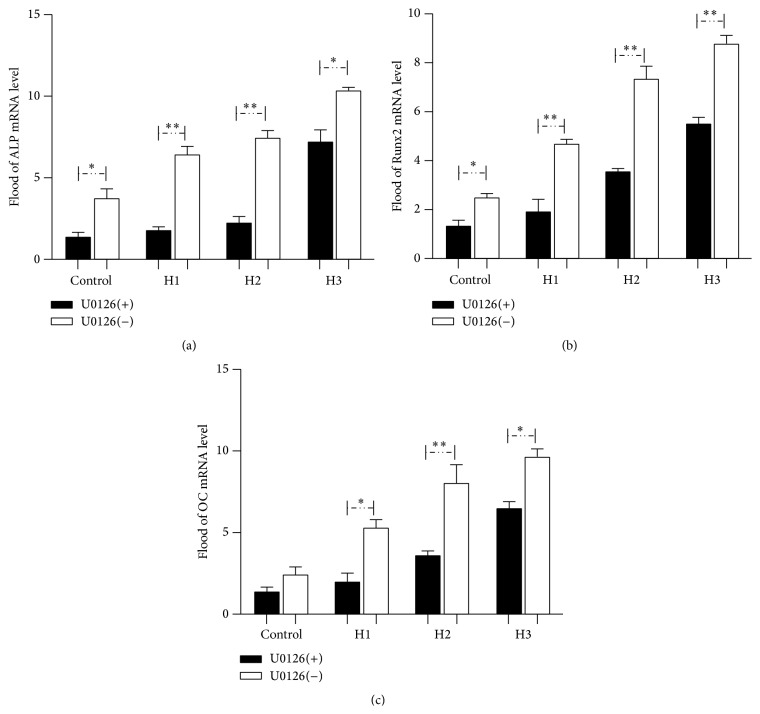
U0126 downregulated the mRNA expression of ALP, Runx2, and OC genes in HBMSCs cultured with or without HAMSCs. Transwells containing HAMSCs were moved into the correlating wells containing HBMSCs in regular medium after cells were attached. After the cells approached confluence in regular medium, both cell types were cultured in OS medium for 7 days, followed by treatment with and without U0126 for a further 24 h. GAPDH was used as the internal control. (a) ALP, (b) Runx2, and (c) OC. ^*∗*^ *P* < 0.05; ^*∗∗*^ *P* < 0.01. Control: HBMSCs cultured without HAMSCs; HBMSCs cocultured with HAMSCs; H1: HBMSC : HAMSC = 1 : 1; H2: HBMSC : HAMSC = 1 : 2; H3: HBMSC : HAMSC = 1 : 3.

Based on the observation that ERK1/2 phosphorylation and Runx2 protein level were inhibited in HBMSCs cultured with HAMSCs in the presence and absence of U0126, we further investigated whether osteogenic differentiation and mineralization in HBMSCs are ERK1/2-dependent.  Figure 7 shows the mRNA expression levels of ALP, Runx2, and OC in HBMSCs cultured with or without HAMSCs in the presence and absence of U0126. All the genes related to OB differentiation and considered to be early HBMSC osteogenic markers were downregulated in the U0126-treated groups in comparison to the levels detected in the untreated groups. Interestingly, the mRNA expression levels were upregulated with the increasing HBMSC : HAMSC ratio with or without U0126 treatment.

**Figure 8:**
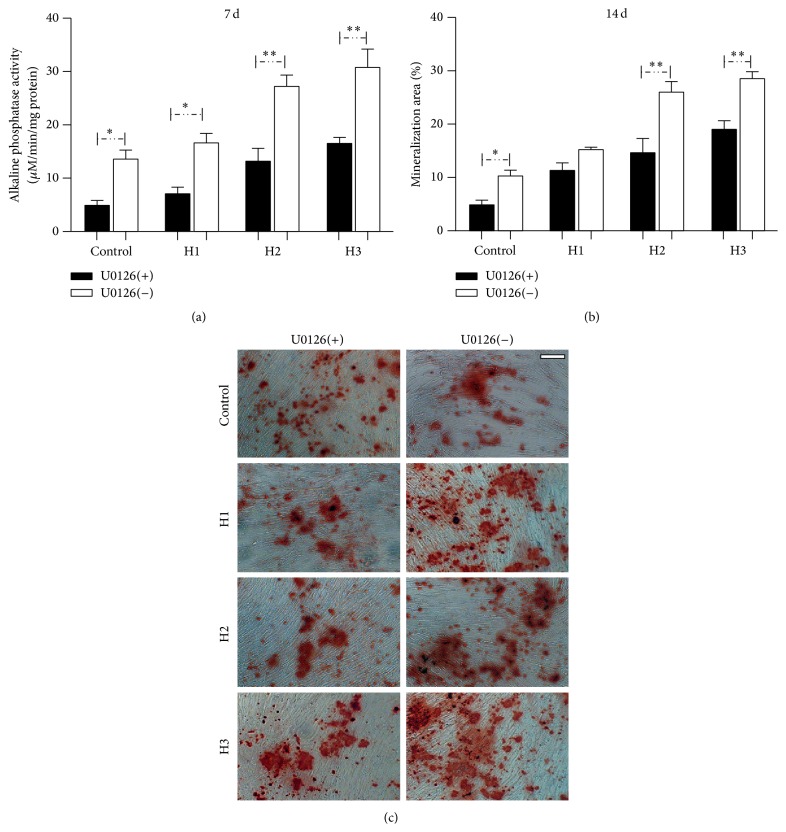
U0126 inhibited the ALP activity and mineralized matrix deposition in HBMSCs cultured with or without HAMSCs. Transwells containing HAMSCs were moved into the correlating wells containing HBMSCs in regular medium after cells were attached. After the cells approached confluence in regular medium, both cell types were cultured in OS medium for 7 days, followed by treatment with and without U0126 for a further 24 h. (a) ALP activity was measured at 7 d using p-nitrophenyl phosphate as the substrate. (b) Mineralized matrix deposition was measured at 14 d by Alizarin red S staining and Image-Pro Plus (IPP) analysis. (c) Micrographs of mineralized matrix deposition, scale bar: 100 *μ*m. ^*∗*^ *P* < 0.05; ^*∗∗*^ *P* < 0.01. Control: HBMSCs cultured without HAMSCs; HBMSCs cocultured with HAMSCs; H1: HBMSC : HAMSC = 1 : 1; H2: HBMSC : HAMSC = 1 : 2; H3: HBMSC : HAMSC = 1 : 3.

To investigate the effect of U0126 on ALP activity, after 7 days in culture with OS medium, the HBMSCs in the control and treatment groups were treated with U0126 for a further 24 h as previously described. The inhibitory effect of U0126 on ALP activity was obvious (Figure 8(a)). As expected, the mineralized matrix deposition by HBMSCs was inhibited by U0126 after 14 days in culture with mineralizing medium and treatment with U0126 for a further 3 days as previously described (Figures 8(b) and 8(c)). Similarly, ALP activity and mineralized matrix formation were upregulated with the increasing HBMSC : HAMSC ratio with or without U0126 treatment.

### 3.6. Discussion

To date, tissue engineering promises to be a potential method for resolving bone deficiency in stomatological diseases. Among all the seed cells, HAMSCs are considered to be an important resource in the field of tissue engineering because of their abundant sources, anti-inflammatory properties, fewer ethical issues, and low immunogenicity [32]. The aim of this study was to find new solutions to the treatment of bone volume inadequacy based on the development of tissue engineering. In this study, HAMSCs were used to promote proliferation and osteoblastic differentiation in HBMSCs. Proliferation levels were investigated by flow cytometry and immunofluorescence staining of EdU, while osteoblastic differentiation and mineralization were evaluated by measuring ALP activity, mineralized matrix formation, and mRNA expression of early HBMSCS osteogenic markers. The findings of the present study support the hypothesis that HAMSCs are capable of providing a preferential environment for driving the proliferation and osteogenic differentiation of HBMSCs and the function of HAMSCs as seed cells in tissue-engineered bone could be derived from its effect on HBMSCs.

To further investigate the mechanism by which osteogenic differentiation of HBMSCs is driven in cocultures with HAMSCs, the activity of the p38, JNK, and ERK1/2 signaling pathways was investigated. The ERK1/2 signaling pathway was shown to play a role in the osteogenic differentiation of HBMSCs, while neither the JNK nor p38 pathways appeared to be involved (data not shown). In this study, activation of ERK1/2 signaling pathway was observed in HBMSCs cocultured with HAMSCs with or without OS. We then used the selective ERK1/2 signaling inhibitor, U0126, to investigate the direct role of the ERK1/2 signaling pathway in the process of HBMSC osteogenic differentiation. As expected, expression of osteogenic differentiation-related genes and mineralized matrix deposition were downregulated by the direct suppression of ERK1/2. This suggests that the ERK1/2 signaling pathway plays a direct and significant role in regulating HBMSC osteogenic differentiation. Interestingly, HBMSCs exposed to HAMSCs and U0126 in combination showed incomplete inhibition in osteogenic differentiation compared with the single-culture groups in the presence of U0126, which indicated that HAMSCs promote osteogenic differentiation in HBMSCs by influencing the ERK1/2 signaling pathway.

The activation of ERK1/2 is also influenced by growth factors [33]. In this study, we found that all the tested indexes gradually increased with the HBMSC : HAMSC ratio, suggesting that the amount of the growth factors responsible for ERK1/2 activation is increased by the presence of a greater proportion of HAMSCs. Previous study has showed that, under the appropriate culture conditions, HAMSC can be induced to various mesenchymal tissues and cells [34]. Furthermore, immunophenotype and RT-PCR analysis showed that HAMSC consistently expressed genes of Oct-4, Rex-1, SCF, NCAM, nestin, BMP-4, GATA-4, HNF-4alpha, vimentin, and so forth [35]. Several of those genes have great potential in osteogenic differentiation [36–38]. Further studies are essential to clarify whether, and to what extent, HAMSCs can influence osteogenic differentiation through these relative genes.

## 4. Conclusions

This study demonstrates the potential of HAMSCs as seed cells in promoting proliferation and osteogenic differentiation of HBMSC by providing a preferential environment for driving these processes. Moreover, we confirmed that activation of the ERK1/2 signaling pathway plays a direct and significant role in regulating HBMSC osteoblastic differentiation. Further investigations of the role of growth factors involved in this system will help clarify the regulatory mechanisms underlying this process and provide support for the application of HAMSCs in the field of tissue engineering.
